# Long-Term Trends in Respiratory Syncytial Virus A Infections (2007–2024) in Korea

**DOI:** 10.3390/diseases13050147

**Published:** 2025-05-10

**Authors:** Jeong Su Han, Sung Hun Jang, Jae-Sik Jeon, Jae Kyung Kim

**Affiliations:** 1 Department of Biomedical Laboratory Science, College of Health Sciences, Dankook University, Cheonan-si 31116, Republic of Korea; jshan1162@naver.com (J.S.H.); zenty87@naver.com (J.-S.J.); 2 Department of Medical Laser, Graduate School of Medicine, Dankook University, Cheonan-si 31116, Republic of Korea; well8143@naver.com

**Keywords:** infant, public health studies, respiratory syncytial virus, seasons, virus diseases

## Abstract

Background/Objectives: Respiratory syncytial virus A (RSV A) is the leading cause of respiratory infections, particularly in vulnerable populations. This study aimed to investigate the long-term epidemiological trends of RSV A infection in the Republic of Korea over an 18-year period (2007–2024), with emphasis on age, sex, and seasonal differences. Methods: A total of 23,284 nasopharyngeal swab specimens were analyzed by multiplex real-time PCR. Statistical comparisons were performed using the chi-square test. Results: The RSV A-positivity rate was highest in 2007 (19.7%) and lowest in 2021 (0.1%) (*p* < 0.001). Infants (0 years) exhibited the highest infection rate (18.5%, 95% CI: 17.3–19.6), whereas adults aged 20–64 years and older adults showed significantly lower rates (0.7% and 0.9%, respectively). Seasonal peaks occurred in winter (15.3%) and autumn (14.7%), indicating earlier onset of RSV A circulation. No significant difference was found between sexes (*p* = 0.196). Conclusions: This study provides the first long-term retrospective analysis of RSV A trends in the Republic of Korea and reveals a shift toward an earlier seasonal onset. These findings support the need for earlier preventive strategies and optimized vaccination timing, particularly for high-risk groups, such as infants. These findings underscore the importance of seasonal variation and the potential influence of environmental factors, such as ambient temperature, relative humidity, and geographic latitude, on RSV A transmission patterns in Korea, although these variables were not directly analyzed in the present study and warrant further investigation.

## 1. Introduction

Respiratory syncytial virus (RSV) is an RNA virus that belongs to the *Orthopneumovirus* genus. It is a major pathogen that causes severe respiratory infections worldwide, particularly in infants, older adults, and immunocompromised individuals [1]. Respiratory syncytial virus A (RSV A) is responsible for an estimated 33 million acute lower respiratory infections worldwide each year, leading to more than 3 million hospitalizations and over 100,000 deaths among children under five years of age [2]. In older adults, RSV A significantly contributes to morbidity and mortality, comparable to seasonal influenza [3]. Despite its global impact, long-term epidemiological data remain limited, particularly in Asian countries such as the Republic of Korea. RSV is classified into two major subtypes, RSV A and RSV B [4], with RSV A being the primary cause of severe lower respiratory tract infections in neonates and infants [5]. RSV infections can lead to complications, such as bronchiolitis and pneumonia, resulting in increased hospitalization and mortality rates, making it a significant public health concern [6].

To date, research on RSV A infections has primarily focused on short-term studies involving infants and young children, with limited long-term analyses of the infection characteristics in young and older adults. Most studies have focused on assessing RSV A infection rates and severity in neonates and children [7]. However, research on how infection rates change with age remains limited. Existing studies on the seasonal prevalence of RSV A infections commonly report peak outbreaks during winter [8]. However, recent studies suggest that the prevalence of RSV A infections may begin to increase in autumn, with seasonal patterns varying by country and region [9]. Nevertheless, these studies are largely based on short-term observations, limiting their ability to comprehensively evaluate long-term variations in RSV A infections. Additionally, limited research has been conducted on how RSV A infection rates changed following the COVID-19 pandemic. During the pandemic, public health interventions, such as social distancing and mask-wearing, reportedly reduced RSV A transmission [10]. However, long-term studies are required to determine whether infection rates have rebounded or are exhibiting new trends.

This study aimed to overcome the limitations of previous short-term studies by comprehensively evaluating the long-term epidemiological changes in RSV A infections using laboratory-confirmed RSV A infection data collected over 18 years (2007–2024) from a single tertiary medical center in the Republic of Korea. Specifically, we analyzed variations in infection rates by age, sex, and season to assess how the pattern of RSV A infections differed across age groups, whether sex was a significant factor influencing infection rates, and whether RSV A exhibited a seasonal pattern that began increasing in autumn, as some recent studies suggest, rather than peaking exclusively in winter. Additionally, we investigated changes in RSV A infection rates following the COVID-19 pandemic to assess the impact of factors such as the immunity gap on RSV A transmission, thus contributing to a more comprehensive understanding of the virus [11]. While most studies on RSV A infections have focused on short-term outbreaks or specific pediatric populations, long-term surveillance data covering all age groups and seasonal patterns are scarce, particularly in the Republic of Korea. This study addresses this gap by analyzing 18 years of laboratory-confirmed RSV A cases, offering comprehensive insights into age-specific, sex-specific, and seasonal trends. We believe that our findings can help refine vaccination and infection prevention strategies [12], inform public health policies, and develop effective measures to protect high-risk populations from RSV A infection [13]. This study aims to analyze trends in RSV A-positivity over an 18-year period (2007–2024) in the Republic of Korea, using laboratory-confirmed data to examine differences by age, sex, and season and to identify early seasonal onset patterns that may inform optimal vaccination timing and public health preparedness.

## 2. Materials and Methods

### 2.1. Study Design

This retrospective observational study utilized molecular diagnostic data from nasopharyngeal swab specimens tested for RSV A at Dankook University Hospital (Cheonan-si, Republic of Korea) during the study period. This study is a retrospective analysis based on laboratory-confirmed RSV A polymerase chain reaction (PCR) test results extracted from the electronic medical records of Dankook University Hospital, a tertiary care facility in the Republic of Korea, from 1 January 2007 to 31 December 2024. The study population included inpatients and outpatients who underwent multiplex real-time PCR testing for respiratory viruses. Due to the retrospective nature of this study and the use of de-identified data, information regarding individual underlying health conditions, immunocompromised status, or specific therapies was not available. Patients who underwent multiplex real-time PCR testing for respiratory viruses at Dankook University Hospital between 1 January 2007 and 31 December 2024, and whose results indicated RSV A detection, were included. Exclusion criteria included incomplete test results or missing essential data, such as sex, age, or test date.

### 2.2. Data Collection

The data were sourced from the multiplex real-time PCR test database of Dankook University Hospital (Cheonan-si, Republic of Korea), covering all tests conducted from 1 January 2007 to 31 December 2024. Patients with incomplete test results or missing essential information, such as sex, age, or test date, were excluded from data collection.

### 2.3. Testing Process

To extract nucleic acids from nasopharyngeal swab specimens, the commercially available QIAamp Viral RNA Mini Kit (Qiagen, Hilden, Germany) was used following the manufacturer’s protocol. The extracted nucleic acids were analyzed using an AdvanSure™ RV-Plus Real-Time RT-PCR Kit (LG Life Sciences, Changwon-si, Republic of Korea), which contains specific primers and probes for detecting RSV A genes. PCR was performed using the SLAN real-time PCR system (LG Life Sciences, Changwon-si, Republic of Korea) with the reaction conditions set according to the manufacturer’s recommendations. The PCR results were interpreted in accordance with the manufacturer’s guidelines for threshold cycle values, ensuring the accurate identification of RSV A-positive samples. According to the manufacturer, detailed information regarding the sensitivity and specificity of the kit was not publicly available.

### 2.4. Data Preprocessing

Data with missing test results were excluded from the analysis. The age groups were classified according to the organizational guidelines of the International Council for Harmonization of Technical Requirements for Pharmaceuticals for Human Use, which is currently used in the Republic of Korea. The classification system included infants (0 years), children (1–19 years), adults (20–64 years), and older adults (≥65 years). The seasonal classification was based on four seasons: spring (March–May), summer (June–August), autumn (September–November), and winter (December–February). Additionally, a year-based categorization was applied to create four analytical variables: age, sex, season, and year.

### 2.5. Data Analysis

Statistical analyses were conducted to examine annual trends in RSV A infection, differences among age groups, sex differences in positivity rates, and seasonal variations. The chi-square (χ^2^) (two-tailed) test was used to assess the statistical significance of the differences between subgroups. The expected values for RSV A-positive and RSV A-negative cases were calculated by multiplying the total number of individuals in each subgroup by the overall RSV A-positivity and -negativity rates, then dividing by the total study population. Statistical significance was set at *p* < 0.05. All statistical analyses were performed using the SPSS software (version 17.0, SPSS Inc., Chicago, IL, USA).

### 2.6. Ethical Considerations

This study was approved by the Institutional Review Board of Dankook University (Approval No. DKU 2025-02-004-003). All personally identifiable information was excluded from this study, and all data were anonymized prior to analysis. Owing to the nature of this study, the requirement for informed consent from the participants was waived.

## 3. Results

### 3.1. Annual Positivity Rate

**Figure 1:**
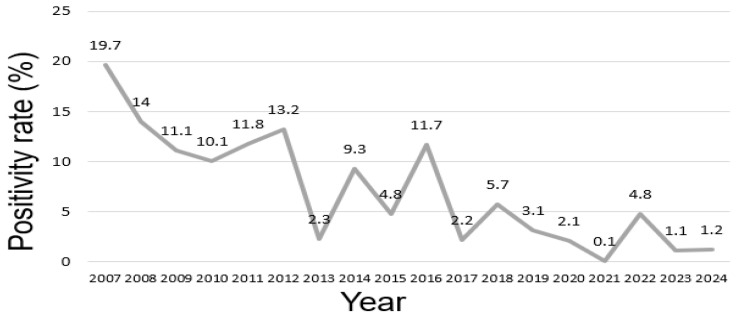
Annual respiratory syncytial virus (RSV) A-positivity rate (%) between 2007 and 2024. The figure presents the annual positivity rates of respiratory syncytial virus A (RSV A) derived from PCR testing of 23,284 nasopharyngeal specimens collected at a single tertiary medical center.

From 2007 to 2024, the RSV A-positivity rate exhibited significant fluctuations, showing an overall decreasing trend (Figure 1). The highest positivity rate was recorded in 2007 (19.7%), whereas the lowest was observed in 2021 (0.1%). A sharp decline occurred between 2012 (13.2%) and 2013 (2.3%), followed by periodic fluctuations. Positivity rates were particularly low in 2020 (2.1%) and 2021 (0.1%), after which a slight increase was observed in 2022 (4.8%). In 2023 (1.1%) and 2024 (1.2%) patients, the positivity rate remained stable.

Statistical analysis revealed that the differences in annual RSV A-positivity rates were statistically significant (χ^2^ = 856.3, χ^2^_0.05,17_ = 27.5, *p* < 0.001). The annual RSV A-positivity rate ranged from a high of 19.7% in 2007 to a low of 0.1% in 2021, indicating substantial fluctuations throughout the study period. Years with sharp declines, such as 2013 and 2021, had a major impact on the overall fluctuations. The absolute number of tests, RSV A-positive cases, and RSV A-negative cases for each year are summarized in Supplementary Table S1. A total of 23,284 tests were performed between 2007 and 2024. The highest number of positive cases was observed in 2007 (209/1057; 19.7%), and the lowest was recorded in 2021 (1/613; 0.1%). However, the period from 2014 to 2016 exhibited relatively stable positivity rates.

### 3.2. Seasonal Positivity Rate

As presented in Table 1, 23,284 specimens collected between 2007 and 2024 were included in this study. To examine seasonal differences in RSV A-positivity rates, analyses were conducted across four seasons: spring (March–May), summer (June–August), autumn (September–November), and winter (December–February). To assess significant differences in positivity rates among seasons, the expected values for each season were calculated and compared with the actual positivity rates, as presented in Table 2.

The analysis revealed that the highest positivity rate was observed in winter (15.3%, 95% CI: 14.4–16.1), followed by autumn (14.7%, 95% CI: 13.7–15.6). In contrast, positivity rates were lower in spring (2.9%, 95% CI: 2.4–3.3) and summer (0.8%, 95% CI: 0.5–1.0) (Table 2). Statistical analysis indicated that the RSV A-positivity rates significantly varied by season, with autumn and winter showing significantly higher positivity rates than spring and summer (*p* < 0.001). These findings suggest that RSV A infections are influenced by seasonal factors, with an increased risk in autumn and winter.

### 3.3. Positivity Rate by Sex

**Figure 2:**
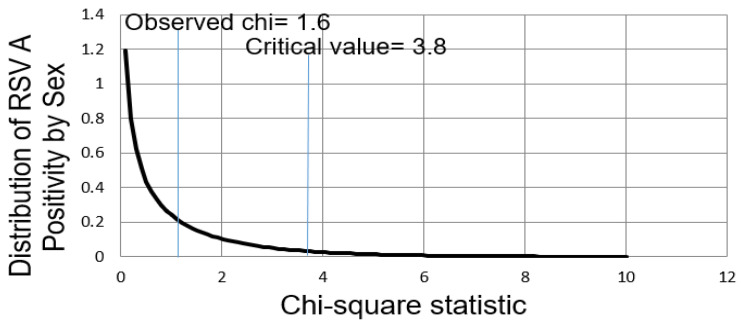
Chi-square distribution for respiratory syncytial virus (RSV) A-positivity by sex. The figure illustrates the distribution of RSV A-positive cases between male and female patients based on PCR results from nasopharyngeal specimens collected from 2007 to 2024.

To evaluate sex-related differences in RSV A infection, 23,284 specimens were analyzed, including 13,961 and 9323 from male and female patients, respectively. The RSV A-positivity rates were 7.5% (95% CI: 7.0–7.9%) in male patients and 8.0% (95% CI: 7.4–8.5%) in female patients, respectively (Table 3). Although the positivity rate was slightly lower in male than in female patients, the difference was not statistically significant (*p* = 0.196) (Figure 2).

The analysis indicated that RSV A infection followed a similar pattern in both male and female patients, suggesting that sex was not a major determinant of the risk of infection. These findings imply that RSV A infection may be influenced more by seasonal factors, age, or other variables than sex.

### 3.4. Positivity Rate by Age Group

**Figure 3:**
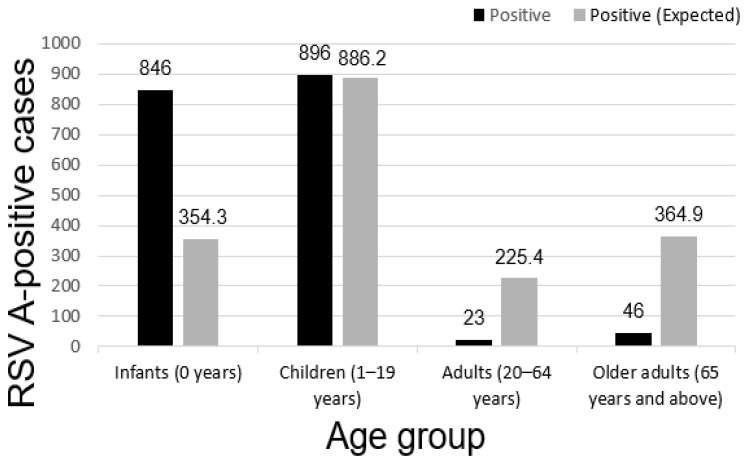
Comparison of observed and expected respiratory syncytial virus (RSV) A-positive cases by age group. The figure compares the observed and expected numbers of RSV A-positive cases across four age groups (infants, children, adults, and older adults) based on long-term surveillance data.

To assess the differences in RSV A infection rates across age groups, the analysis was conducted by categorizing individuals into infants (0 years) (95% CI: 17.3–19.6), children (1–19 years) (95% CI: 7.5–8.5), adults (20–64 years) (95% CI: 0.4–1.0), and older adults (≥65 years) (95% CI: 0.6–1.1) (Table 4). The observed positive cases were compared to the expected values (Table 5). Figure 3 illustrates the differences between the observed and expected RSV A-positive cases for each age group.

In the infant (0 years) group, the observed number of positive cases (846 cases) significantly exceeded the expected value (354.3 cases), indicating a substantially higher RSV A infection rate than anticipated. In contrast, the adult (20–64 years) group showed a remarkably lower actual positivity count (23 cases) compared to the expected value (225.4 cases), suggesting a relatively lower RSV A infection rate in this age group.

Statistical analysis confirmed that the differences in RSV A-positivity rates across age groups were statistically significant (*p* < 0.001), with the most notable disparities observed between the infant (0 years) group and both the adult (20–64 years) and older adult (≥65 years) groups (Figure 3). These findings highlight age as a critical factor influencing the risk of RSV A infection and emphasize the need for targeted prevention strategies, particularly in the infant population.

## 4. Discussion

This study involved a long-term retrospective analysis of RSV A-positivity rate variations over 18 years (2007–2024), confirming that the differences in annual positivity rates were statistically significant (χ^2^ = 856.3, χ^2^_0.05,17_ = 27.5, *p* < 0.001). These findings suggest that epidemiological fluctuations in RSV A are influenced by viral evolution, environmental factors, and public health interventions. The sharp decline in RSV A-positivity between 2012 and 2013 may be related to viral circulation patterns, changes in herd immunity, or modifications in surveillance systems. Additionally, the significant decrease in positivity rates during 2020–2021 was likely a result of widespread public health interventions implemented during the COVID-19 pandemic, which effectively suppressed RSV transmission [14,15]. The gradual increase in positivity rates from 2022 and the stabilization observed in 2023–2024 may be attributed to the relaxation of public health measures and the phenomenon of the immunity gap [16]. A seasonal analysis of 23,284 specimens collected between 2007 and 2024 revealed significant differences in RSV A-positivity rates across seasons. The highest positivity rates were observed in the winter (15.3%) and autumn (14.7%), whereas the rates were significantly lower in the spring (2.9%) and summer (0.8%). These differences were statistically significant (*p* < 0.001), indicating that RSV A infection is strongly influenced by seasonal factors.

Previous studies have reported that RSV A infections sharply peak in winter [17]. However, this study presents a key finding that differs from existing research: the prevalence of RSV A infections began to significantly increase in autumn (September–November), earlier than expected. While most studies have identified winter as the primary epidemic period for RSV A [18], this study found a considerable infection rate (14.7%) in autumn. These results suggest that RSV A is not solely a winter-season virus but rather follows a pattern of gradual increase in autumn, peaking in winter. The autumn increase and winter peak in RSV A activity highlight the need for earlier public health preparedness, including timely surveillance and potential vaccine deployment prior to the peak season. This underscores the necessity to strengthen infection prevention and surveillance measures earlier in the year. Our observation that RSV A activity increased during autumn and peaked in winter aligns with the findings from other temperate regions. For instance, in the United States, RSV epidemics traditionally began in October and peaked in December, although the COVID-19 pandemic caused shifts in these patterns [19]. Similarly, studies from Italy have reported winter peaks in RSV activity [20].

Regarding age-specific susceptibility, our data, which indicate the highest positivity rates among infants under 1 year of age, are consistent with global trends. A study analyzing the global burden of RSV reported that children under 5 years old, particularly those under 6 months, face the highest risk of severe RSV infection [2]. This study expands the traditional hypothesis regarding the seasonal patterns of RSV A infection and provides valuable insights for infection prevention and public health policy development. The observed increase in the prevalence of RSV A infections during the autumn suggests that preventive measures should be implemented earlier than previously planned.

With the ongoing development of RSV vaccines, optimizing the vaccination timing has become a critical issue [21,22]. The findings of this study provide essential evidence for formulating RSV prevention strategies, suggesting that adjusting vaccination schedules in autumn may be an effective infection control strategy. Additionally, this study offers significant implications for healthcare system preparedness, emphasizing the need for early allocation of medical resources, expansion of diagnostic testing, and protective measures for high-risk groups (infants, older adults, and individuals with chronic conditions). The finding that the prevalence of RSV A infections began to increase in autumn rather than exclusively in winter highlights the need for early medical preparedness and resource distribution.

This study also analyzed a large dataset of RSV A infections from 2007 to 2024 and found that there were no statistically significant differences in RSV A-positivity rates between male and female patients (*p* = 0.196). Although the positivity rate was slightly higher in females, the difference was not statistically significant. This suggests that sex may not be a major determinant of RSV A infection risk in this population, which contrasts with some reports that show male predominance in severe RSV cases. This indicates that RSV A infection was likely influenced more by environmental factors, such as season, and intrinsic factors, such as age, than by sex. While previous studies have reported inconsistent conclusions regarding the impact of sex on RSV infections [23,24], this study provides robust evidence from a long-term dataset, confirming that sex is not a major risk factor for RSV A infection. This finding contributes to academic knowledge by demonstrating that age-based rather than sex-difference-based approaches are more effective for RSV prevention and vaccination strategies, with a strong emphasis on protecting high-risk groups such as infants and immunocompromised individuals [25]. Although this study found no statistically significant difference in RSV A-positivity rates between men and women, a sex- and gender-based analysis may still offer valuable insights. Biological factors, such as hormonal influences on immune responses, and social determinants, including differences in exposure risks or health-seeking behavior, could contribute to variations in susceptibility and disease outcomes. Future studies incorporating sex- and gender-based analysis are warranted to better understand the complex interactions between sex, gender, and viral infections such as RSV A.

The age-based analysis of RSV A infection rates confirmed significant statistical differences among age groups (*p* < 0.001), with the infant (0 years) group exhibiting a substantially higher-than-expected positivity rate, while adults (20–64 years) and older adults (≥65 years) showed lower-than-expected positivity rates. These findings highlight age as a critical factor influencing RSV A infection risk, emphasizing the need for targeted preventive strategies for infants. The particularly high positivity rate in infants (18.5%) reflects their immature immune system and limited exposure to RSV, making them highly susceptible. This finding aligns with previous global reports and underscores the importance of prioritizing this group for future RSV vaccine interventions. Another discovery in this study was that the differences in RSV A infection rates across age groups were not random variations but rather resulted from biological and environmental factors, such as immune development, social behavior, and exposure patterns. The significantly higher infection rate in infants reaffirms that this age group is the most vulnerable to RSV A infection [26]. From a public health perspective, this underscores the necessity for dedicated prevention strategies for infants, including early vaccination and infection control measures. The lower expected infection rates in younger and older adults suggest that repeated exposure to RSV A may contribute to partial immunity, which is an important consideration for future RSV vaccine development and immune response research [27]. These findings align with those of previous studies, which reported that infants have an immature immune system, making them highly susceptible to RSV A infections. Additionally, they have a higher likelihood of progressing to severe disease, particularly when maternal passive immunity declines [28]. By contrast, adults develop immunity through repeated RSV exposure, thereby reducing their susceptibility to infection. Three factors may explain the observed differences in RSV A infection rates by age group: (1) Immune system development—infants have weaker immune defenses against RSV A, while adults and older adults may have acquired partial immunity through repeated exposures. (2) Social behavior and exposure frequency—infants and children have higher exposure risks in daycare and school environments [29], whereas adults may be exposed to RSV A in workplaces but benefit from prior immune responses, leading to lower infection rates. Additionally, older adults may have reduced social interactions, limiting their exposure to RSV A. (3) Limited preventive measures for infants—the absence of an approved RSV A vaccine for infants may contribute to their higher infection rates, highlighting the need for enhanced prevention strategies. This study provides quantitative insights into age-related differences in RSV A infection, confirming that RSV A infection rates significantly differ by age group. This study is one of the first comprehensive, long-term analyses of RSV A epidemiology in the Republic of Korea, identifying consistent seasonal peaks and high positivity rates in infants. These insights contribute to the understanding of the RSV A burden in temperate Asian regions. Moreover, the findings have practical implications for surveillance and public health policy, including the timing of resource allocation, development of early warning systems, and consideration of RSV vaccination schedules for vulnerable populations.

Using chi-square analysis to compare the observed and expected values, this study validated its findings against those of the existing literature. Furthermore, by analyzing long-term RSV A infection data, this study provides a clear understanding of infection patterns and their implications for public health interventions. The results emphasize that infants are the most vulnerable age group, requiring early preventive measures, while in younger and older adults, the focus should be on managing severe cases rather than preventing the infection itself [30,31]. Future research should include multicenter or multinational studies that incorporate various climatic and geographical factors to provide a broader perspective on RSV A epidemiology. Further studies should integrate meteorological data and viral genetic variation analysis to gain a deeper understanding of seasonal RSV A dynamics and determine whether specific RSV A lineages dominate and impact transmission patterns [32,33]. Moreover, additional research should focus on the clinical burden (hospitalization rates, intensive care unit admissions, and mortality), external environmental factors (air pollution and climate change), and interactions between RSV A and RSV B [34].

To the best of our knowledge, this is one of the most comprehensive long-term retrospective analyses of RSV A epidemiology in the Republic of Korea. Unlike previous studies, which primarily focused on short-term trends, this study provides a holistic view of RSV A outbreaks over 18 years. These findings highlight a long-term decline in RSV A-positivity rates, suggesting possible changes in transmission patterns and herd immunity levels. Additionally, the post-pandemic resurgence of RSV A infections was interpreted in relation to the immunity gap, thereby offering valuable epidemiological insights. Future research should incorporate underlying health conditions, vaccination status, seasonal factors, and regional variations to further refine our understanding of RSV A infection across different populations. According to the recent literature, the pathogenesis of RSV involves multiple interrelated mechanisms, including disruption of the airway epithelial barrier, neurogenic inflammation, and immune dysregulation. Injury to the epithelial cells increases mucosal permeability, facilitates the penetration of allergens and pathogens, and promotes airway remodeling, a process that has been implicated in the development of chronic wheezing and asthma, particularly following infections during early infancy. Neurogenic inflammation, mediated by neuropeptides such as substance P, further exacerbates airway hyperresponsiveness and local inflammation. Additionally, RSV modulates host immunity by inducing cytokines such as interleukin-13 (IL-13), which drive a type 2-skewed immune response and suppress effective antiviral defenses. These pathogenic pathways are closely aligned with our epidemiological finding of a markedly high RSV A-positivity rate in infants, suggesting that primary RSV A infection during early infancy may predispose this vulnerable population to long-term pulmonary complications [35].

In summary, this study offers one of the most comprehensive, long-term epidemiological analyses of RSV A in the Republic of Korea, providing 18 years of surveillance data that reveal consistent seasonal trends and a disproportionately high burden among infants under one year of age. These findings expand our regional understanding of RSV A transmission dynamics in temperate Asian settings and underscore the need for timely public health responses. From a policy perspective, our results support the development of age-targeted RSV vaccination strategies, particularly for infants and other high-risk groups, and suggest that vaccination campaigns or prophylactic interventions should be timed before the onset of autumn. In addition, this study highlights the importance of enhancing Korea’s RSV surveillance infrastructure through early warning systems, real-time reporting, and integration with pediatric care services. Future research should explore the genetic diversity of circulating RSV strains, evaluate post-introduction vaccine performance, and investigate host and environmental factors that may influence transmission and clinical severity across different populations.

## 5. Limitations

This study has several limitations. First, as it was conducted at a single tertiary medical institution, the findings may not be fully generalizable to the nationwide epidemiology of RSV A infections in the Republic of Korea. Second, due to its retrospective design, this study did not account for important clinical and environmental variables such as disease severity, underlying medical conditions, vaccination status, temperature, humidity, population mobility, and co-infections, all of which may have influenced RSV A-positivity rates. Third, we did not perform genetic lineage analyses of RSV A, despite the circulation of multiple genotypes (e.g., ON1 and GA2) that may differ in transmissibility, virulence, and immune evasion capacity. Fourth, laboratory procedures, including test indications, specimen transport conditions, and PCR kit versions or protocols, evolved over the 18-year study period. These methodological variations may have introduced temporal bias or led to case misclassification. Finally, environmental, behavioral, and socioeconomic factors (e.g., climate, population density, and healthcare accessibility) were not assessed, limiting our ability to adjust for potential confounders affecting RSV transmission and detection.

## 6. Conclusions

In conclusion, this study analyzed RSV A infection data from 2007 to 2024, evaluating variations by age, sex, and season. The findings confirmed that the risk of RSV A infection was significantly influenced by age and seasonal factors, with the highest infection rates observed in infants and lower rates in adults and older adults. This suggests that prevention strategies should prioritize infants, while managing disease severity may be more important for both adults and older adults. Seasonal variations showed higher positivity rates in winter and autumn, indicating an earlier increase in RSV A infections, emphasizing the need for earlier preventive measures. No significant sex differences in the infection rates were observed. As one of the largest retrospective analyses of RSV A epidemiology, this study provides valuable insights for public health policies, highlighting the importance of age-specific prevention and vaccination strategies and considering infection timing for optimal intervention. The findings of this study can serve as a critical foundation for future clinical research, particularly in determining the optimal age and timing of RSV vaccination as well as in guiding the development of RSV therapeutics and infection control measures.

## Figures and Tables

**Table 1:** RSV A seasonal positivity rates from 2007 to 2024.

Season	Total	Positive	Negative	Positivity Rate (%)
Spring	6391	189	6202	2.9
Summer	4810	41	4769	0.8
Autumn	5607	719	4888	14.7
Winter	6476	862	5614	15.3

The positivity rate was calculated as follows: (positive/[positive + negative]) × 100. RSV, respiratory syncytial virus.

**Table 2:** Expected RSV A seasonal positive and negative values from 2007 to 2024.

Season	Positive Expected	Negative Expected
Spring	14.5	6376.5
Summer	3.1	4806.9
Autumn	55.3	5551.7
Winter	66.3	6409.7

Expected values were calculated for independence using the chi-square test assumptions. RSV, respiratory syncytial virus.

**Table 3:** Distribution of respiratory syncytial virus (RSV) A-positive and -negative cases and positivity rates by sex.

Sex	Total	Positive	Negative	Positivity Rate (%)
Male	13,961	1060	12,901	7.5
Female	9323	751	8572	8.0

The positivity rate was calculated as follows: (positive/[positive + negative]) × 100. No statistically significant difference was observed between the sexes (*p* = 0.196). RSV, respiratory syncytial virus.

**Table 4:** Distribution of the RSV A-positivity rates by age group from 2007 to 2024.

Age Group	Total	Positive	Negative	Positivity Rate (%)
Infants (0 years)	4556	846	3710	18.5
Children (1–19 years)	11,137	896	10,241	8.0
Adults (20–64 years)	2899	23	2876	0.7
Older adults (≥65 years)	4692	46	4646	0.9

The positivity rate was calculated as follows: (positive/[positive + negative]) × 100. RSV, respiratory syncytial virus.

**Table 5:** Expected RSV A cases by age group.

Age Group	Positive (Expected)	Negative (Expected)
Infants (0 years)	354.3	4201.6
Children (1–19 years)	886.2	10,270.7
Adults (20–64 years)	225.4	2673.5
Older adults (≥65 years)	364.9	4327.0

Expected values were calculated for independence using the chi-square test assumptions. RSV, respiratory syncytial virus.

## Data Availability

Data supporting the findings of this study are not publicly available because of privacy and ethical restrictions. The dataset contained sensitive results and was used with the approval of the Institutional Review Board (IRB) of Dankook University Hospital. Requests for data access may be considered only under reasonable circumstances and must be approved by both the corresponding author (supervising professor) and the IRB.

## Supplementary Materials

The following supporting information can be downloaded at: https://www.mdpi.com/article/10.3390/diseases13050147/s1, Supplementary Table S1. Annual number of RSV A tests, negative and positive cases, and positivity rates from 2007 to 2024.

## Abbreviations

The following abbreviations are used in this manuscript:

RSV	respiratory syncytial virus
RT-PCR	real-time polymerase chain reaction
