# Supplementary material for: Long-Term Trends in Respiratory Syncytial Virus A Infections (2007–2024) in Korea
Source: Diseases. 2025 May 10;13(5):147. doi: 10.3390/diseases13050147 (PMC12110253; doi:10.3390/diseases13050147)
Supplement: Supplementary file 1 [file diseases-13-00147-s001.zip › diseases-3592222-supplementary.pdf]

Supplementary Table S1. Annual number of RSV A tests, negative and positive cases, and positivity rates from 2007 to 2024.

This table presents the absolute number of tests performed each year, the number of RSV A-positive and -negative results, and the corresponding positivity rates (%).

Year	Total	Negative	Positive	Positivity rate (%)
2007	1,057	848	209	19.7
2008	1,504	1,293	211	14
2009	1,265	1,124	141	11.1
2010	1,657	1,488	169	10.1
2011	1,568	1,382	186	11.8
2012	1,345	1,167	178	13.2
2013	1,545	1,508	37	2.3
2014	1,674	1,518	156	9.3
2015	1,388	1,320	68	4.8
2016	1,645	1,452	193	11.7
2017	1,436	1,404	32	2.2
2018	1,834	1,728	106	5.7
2019	1,432	1,387	45	3.1
2020	792	775	17	2.1
2021	613	612	1	0.1
2022	860	818	42	4.8
2023	1,016	1,004	12	1.1
2024	653	645	8	1.2
